# Letter to the Editor: “Continuous use of metformin in patients receiving contrast medium: What is the evidence? A systematic review and meta-analysis”

**DOI:** 10.1007/s00330-024-10862-w

**Published:** 2024-07-09

**Authors:** Yanan Ge, Lingping Ma, Hua Luo

**Affiliations:** 1 Department of Ultrasound, Taizhou Second People’s Hospital, Taizhou, Zhejiang China; 2 Department of Operation Room, Taizhou Hospital of Zhejiang Province Affiliated to Wenzhou Medical University, Taizhou, Zhejiang China; 3 Department of Orthopedics, Taizhou Hospital of Zhejiang Province Affiliated to Wenzhou Medical University, Taizhou, Zhejiang China

We have a great interest in the systematic review and meta-analysis titled “Continuous use of metformin in patients receiving contrast medium: What is the evidence? A systematic review and meta-analysis” by Kao et al [1]. In their study, they included a total of six studies (two RCTs and four retrospective cohort studies) comprising 1459 patients [2–7]. The results of the study indicated that continuing the use of metformin did not increase the risk of contrast-induced acute kidney injury, lactic acidosis, or deterioration in renal function compared to patients who discontinued metformin or were not metformin users. We sincerely appreciate the efforts of the authors in data collection and analysis. This study supports the argument that there is no need to discontinue metformin before contrast medium administration in patients with eGFR greater than 30 mL/min/1.73 m², providing valuable guidance for healthcare professionals. However, while reviewing the article, we noticed several issues that we would like to address:

Firstly, the abstract of the article stated that the purpose of the study was to investigate the effect of metformin in diabetic patients receiving contrast medium treatment. Therefore, we believe that the study population should have been diabetic patients. However, the search keywords and inclusion/exclusion criteria did not explicitly specify diabetic patients. Although this does not affect the conclusions of the article, we believe it reflects a lack of rigour in this aspect.

Secondly, the article excluded non-English literature, which could potentially introduce publication bias. We suggest that this limitation should be detailed in the article to assist readers in evaluating the reliability of the conclusions.

Thirdly, the study included Lal et al’s research [7], which we noticed was published in the form of a comment. Comment articles may not have undergone peer review or scrutiny, and their shorter length may result in only partial reporting of research results rather than a complete dataset. This may introduce publication bias and affect the results of the meta-analysis.

Fourthly, the article combined the results of RCTs and non-RCTs for analysis, which is uncommon in meta-analyses due to the significantly different risk of bias between the two types of studies [8]. We believe that separate analyses should be conducted for RCTs and non-RCTs to improve the reliability and interpretability of the results.

**Fig. 1:**
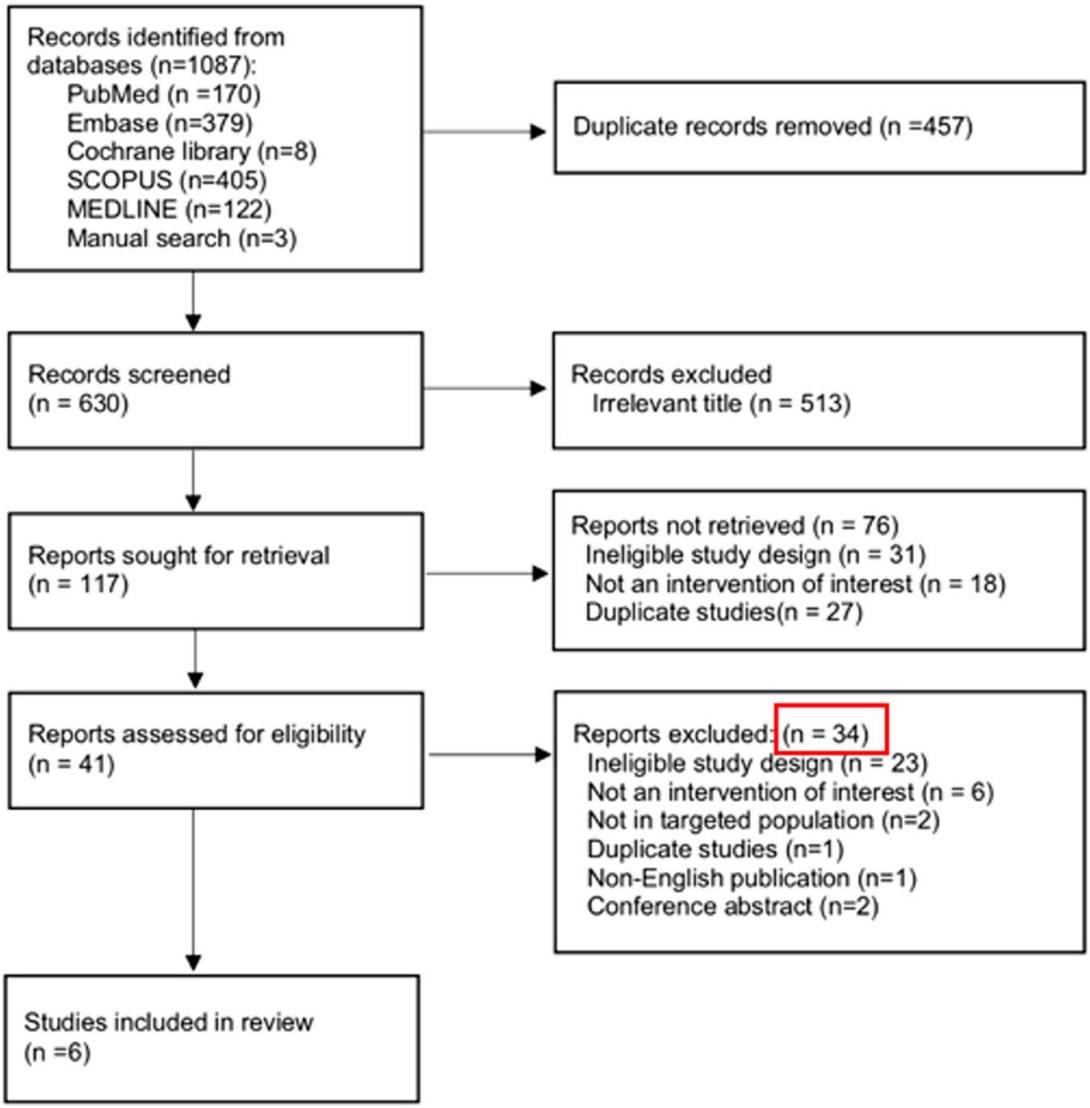
Detailed search flow reproduced with permission from Kao et al [1]. We indicated the data with errors using the red box

Lastly, as the detailed search flow shown in Fig. 1, we observed that the number of reports excluded after screening for eligibility should be 35, not 34. The authors noted in the figure that ineligible study design (*n* = 23); not an intervention of interest (*n* = 6); not in the targeted population (*n* = 2); duplicate studies (*n* = 1); non-English publication (*n* = 1); conference abstract (*n* = 2). Adding these numbers together should result in 35 excluded reports, not 34. As indicated in Fig. 1, we have marked the relevant sections with red boxes.

In conclusion, we commend the authors for their outstanding work. It is our aspiration that our correspondence will provide readers with further insights into this topic.
